# Synthesis and structure of 3-methylpyridinium 3-carboxy-5-nitrobenzoate monohydrate

**DOI:** 10.1107/S2056989026008091

**Published:** 2026-08-11

**Authors:** Karmajyoti Borah, Sourajyoti Ray, Sanchay Jyoti Bora

**Affiliations:** a Department of Chemistry, Pandu College, Guwahati-781012, Assam, India; b Department of Chemistry Gauhati University,Guwahati-781014 Assam India; Indian Institute of Science Education and Research Bhopal, India

**Keywords:** crystal structure, co-crystal, supra­molecular inter­actions, hydrogen bonding

## Abstract

The title compound, C_6_H_8_N^+^·C_8_H_4_NO_6_^−^·H_2_O, crystallizes in the monoclinic crystal space group *C*2/*c*. An N—H⋯O hydrogen bond is observed between the 3-methyl­pyridinium cation and the 5-nitro­isophthalate anion. The crystal packing is consolidated by an extensive network of inter­molecular O—H⋯O, N—H⋯O, and C—H⋯O hydrogen bonds, further reinforced by π–π inter­actions.

## Abstract 2

A hydrated salt of 3-methyl­pyridine and 5-nitro­isophthalic acid has been synthesized using the reflux method. The crystal structure is consolidated by a network of significant inter­molecular inter­actions, including O—H⋯O, N—H⋯O, and C—H⋯O hydrogen bonds, along with additional π–π inter­actions, which collectively contribute to the robustness of the solid-state architecture.

## 1. Chemical context

Co-crystals are homogeneous crystalline solids composed of two or more neutral components in definite stoichiometric ratios, assembled under ambient conditions through non-covalent inter­actions such as hydrogen bonding, π–π stacking, and van der Waals forces (Pan *et al.*, 2019 ▸). The key distinction between a co-crystal and a salt lies in the position of the acidic proton within the acid–base pair. When proton transfer to the base is complete, salt formation is favoured over co-crystal formation. The acidity of aromatic acids, and hence their proton-donating ability, is strongly influenced by the nature and position of substituents on the aromatic ring. Electron-withdrawing groups tend to enhance acidity, whereas electron-donating groups reduce it. Consequently, the tendency of a hydrogen-bond acceptor to form either a co-crystal or a salt depends significantly on these substituent effects.

Previous studies by Mohamed *et al.* (2009 ▸) have highlighted the role of pyridine–carb­oxy­lic acid and pyridinium–carboxyl­ate synthons in governing the crystal packing of binary solid systems. In addition, our group has reported co-crystals of gallic acid with 4-cyano­pyridine, demonstrating the significance of O—H⋯N inter­molecular hydrogen bonding in consolidating the structure (Goswami *et al.*, 2021 ▸). Another study from our group describes the crystal structure of phenyl­enedi­acetic acid with 4,4′-bi­pyridine, where similar hydrogen-bonding motifs, including O—H⋯N, C—H⋯N, and C—H⋯π inter­actions, play a crucial role in consolidating the solid-state architecture (Paul & Bora, 2015 ▸).
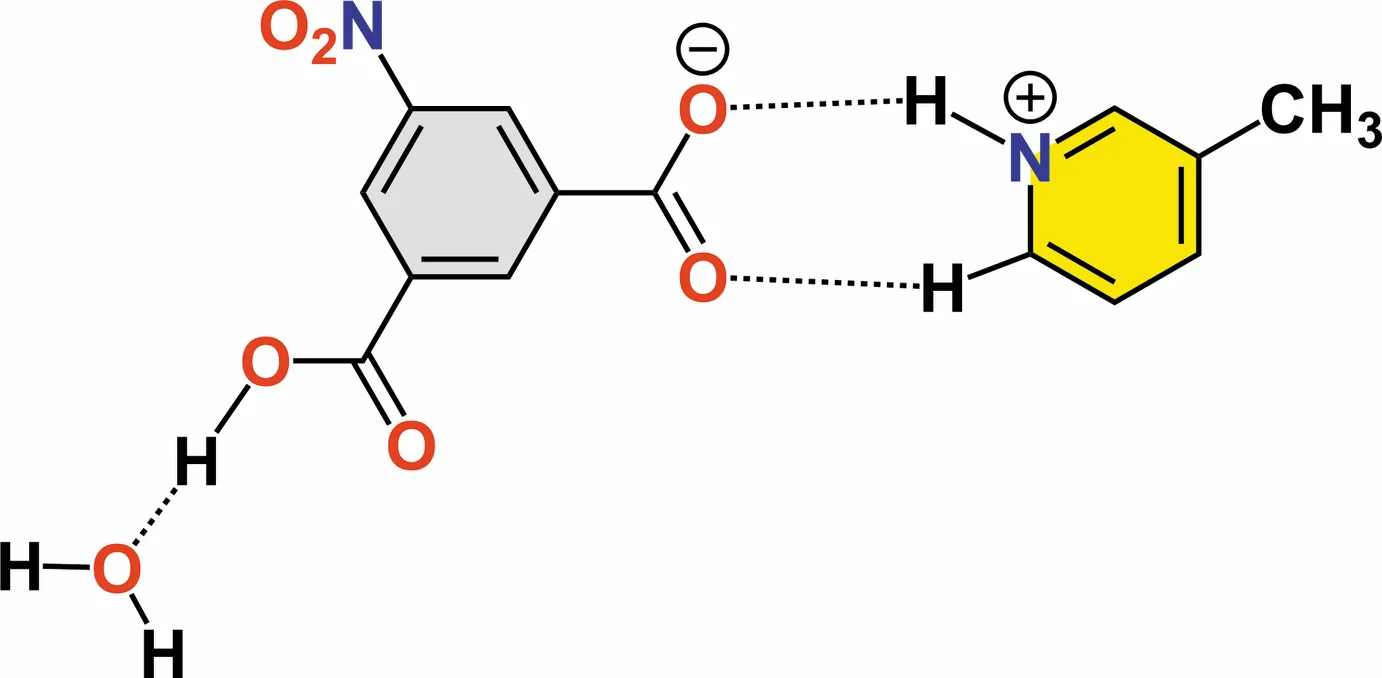


## 2. Structural commentary

**Figure 1:**
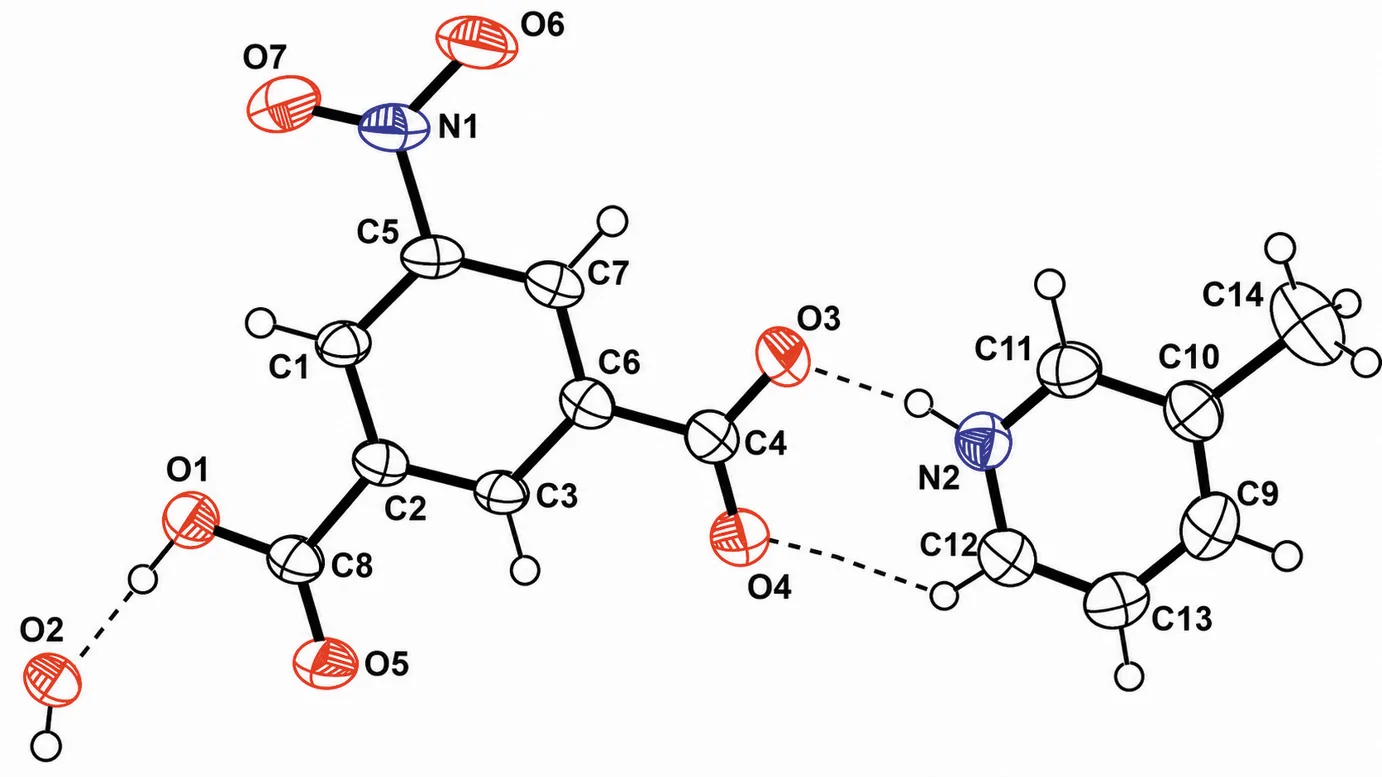
The mol­ecular structure of the title compound with displacement ellipsoids drawn at 50% probability. All the non H-atoms are labelled. The dashed lines depict hydrogen bonds.

The title hydrated salt (Fig. 1 ▸) crystallizes in the monoclinic crystal system with the space group *C*2/*c*. In this structure, 3-methyl­pyridine accepts a proton from 5-nitro­isophthalic acid, resulting in the formation of a pyridinium cation. The protonated base and the corresponding carboxyl­ate anion together constitute a salt, consolidated by N—H⋯O and C—H⋯O hydrogen bonds, which can be described by the graph-set motif 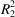
(7). In addition, the water mol­ecule of crystallization participates in an extended hydrogen-bonding network with the 5-nitro­isophthalate moiety, further reinforcing the supra­molecular assembly. Analysis of the difference-Fourier map reveals positive electron density near the nitro­gen atom (N2) at a distance of 0.88 (3) Å, consistent with a typical N*sp*^2^—H bond length. Additionally, a negative residual electron density peak is observed near the O3 atom, indicating proton transfer between the acid and pyridine components. Consequently, the pyridinium cation is consolidated by the carboxyl­ate anion through strong hydrogen bonding, leading to the formulation of the compound as a hydrated salt, C_6_H_8_N^+^C_8_H_4_NO_6_·H_2_O.

## 3. Supra­molecular features

**Figure 2:**
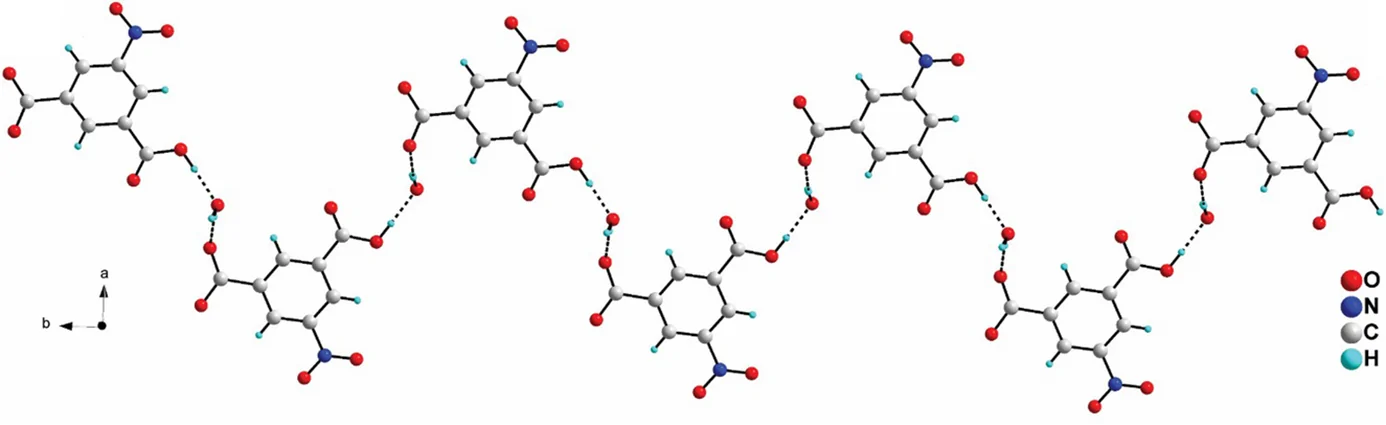
Water-mediated O—H⋯O linkage showing the chain structure.

**Figure 3:**
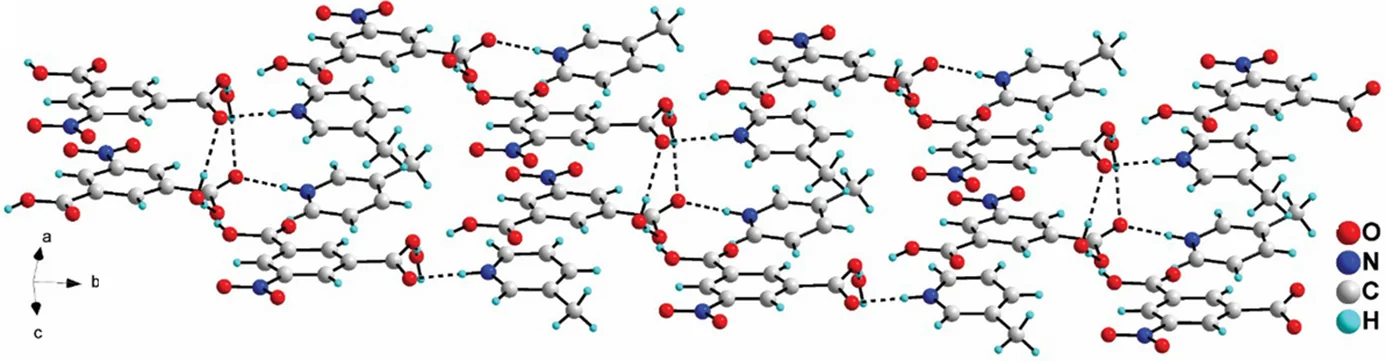
Hydrogen-bonded layer viewed down the *bc* plane.

**Figure 4:**
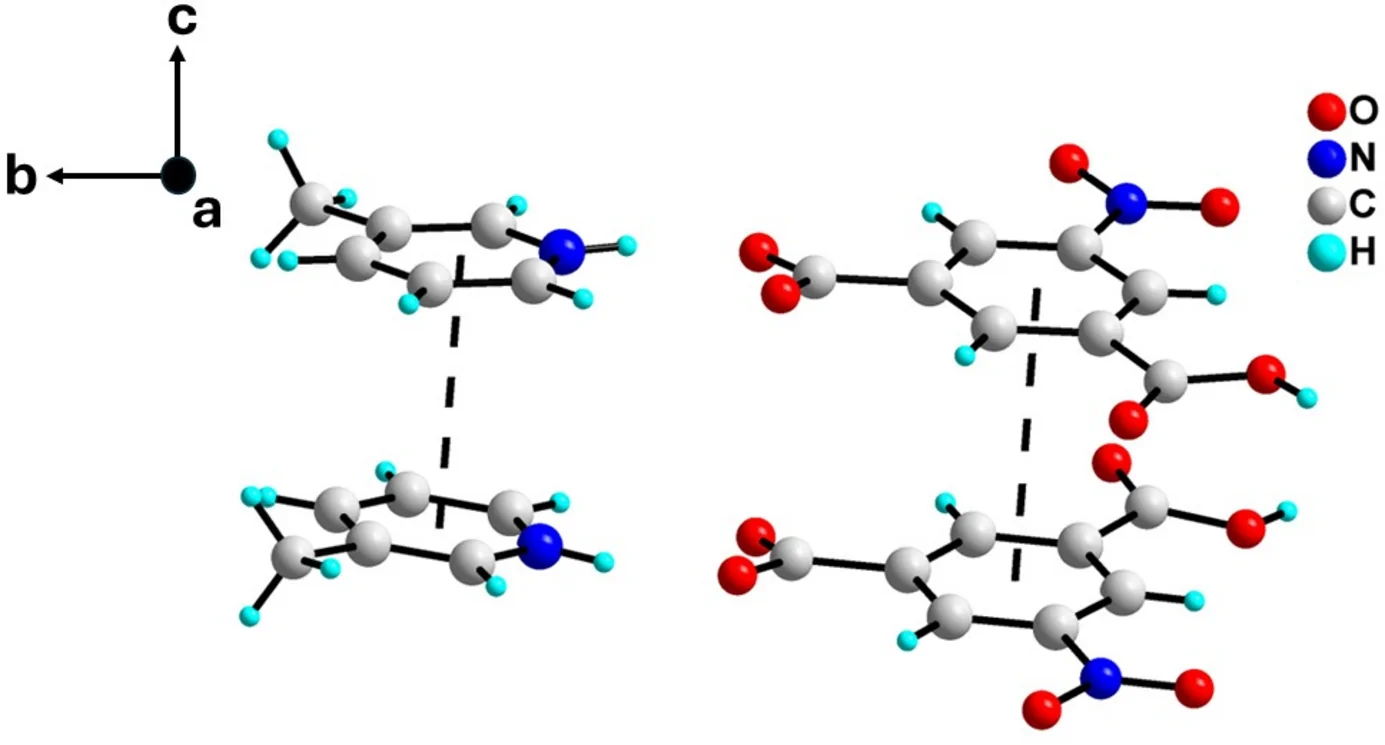
π–π stacking inter­actions between pyridinium and phenyl rings viewed down the *bc* plane

The water mol­ecule of crystallization further acts as a hydrogen-bond donor through the O2—H2*A*⋯O4 inter­action [O⋯O = 2.718 (2) Å], linking neighbouring carboxyl­ate groups belonging to adjacent 5-nitro­isophthalate units. Consequently, the water mol­ecule functions as a supra­molecular bridge, propagating the structure into an extended hydrogen-bonded chain running through the crystal structure (Table 1 ▸, Fig. 2 ▸). The hydrogen-bonded chains are further inter­connected through electrostatic inter­actions between the protonated 3-methyl­pyridinium cations and the negatively charged 5-nitro­isophthalate anions. Additional weak C—H⋯O contacts involving pyridinium and aromatic C—H donors with carboxyl­ate and nitro oxygen atom acceptors reinforce these connections, leading to the formation of extended supra­molecular sheets (Fig. 3 ▸). Beyond hydrogen bonding, the crystal packing is further consolidated by aromatic π–π stacking inter­actions between neighbouring pyridinium and benzene rings [*Cg*1⋯*Cg*1(1 − *x*, *y*,1 /2 − *z*) = 3.7963 (14) Å and *Cg*2⋯*Cg*2 = 3.5924 (15) Å where *Cg*1 and *Cg*2 are the centroids of the C1–C3/C5–C7 and N2/C9–C13 rings, respectively]. These inter­actions promote the formation of columnar arrangements along specific crystallographic directions and facilitate efficient mol­ecular packing (Fig. 4 ▸). The combined effect of strong O—H⋯O hydrogen bonds, water-mediated bridges, weak C—H⋯O inter­actions and π–π inter­actions ultimately generates a highly inter­connected three-dimensional network. Thus, the crystal structure may be viewed as a hydrogen-bond-directed framework in which water mol­ecules of crystallization play a central structure-directing role, while aromatic stacking inter­actions provide additional consolidation and packing efficiency.

Table 1 ▸ summarizes the hydrogen-bonding parameters. The crystal structure is primarily consolidated by a network of strong and highly directional O—H⋯O and N—H⋯O hydrogen bonds, with additional contributions from weaker C—H⋯O inter­actions. The O1—H1⋯O2 and N2—H2⋯O3 hydrogen bonds exhibit short H⋯*A* distances [1.64 (3) and 1.77 (3) Å] and nearly linear bond angles [170 (3) and 171 (3)°], indicating their significant role in enhancing the crystal cohesion. Similarly, the O2—H2*A*⋯O4 inter­action displays a near-linear geometry [171 (3)°] with a short H⋯*A* distance [1.89 (2) Å], further strengthening the supra­molecular framework. In contrast, the O2—H2*B*⋯O3 hydrogen bond is relatively weaker, as evidenced by its longer H⋯*A* distance [2.03 (2) Å] and slightly reduced bond angle [163 (3)°]. The weakest inter­action in the structure is the C12—H12⋯O4 contact, characterized by a comparatively long H⋯*A* distance (2.59 Å) and a markedly bent geometry (124°), typical of weak C—H⋯O inter­actions.

Thus, the crystal structure develops through a stepwise assembly process: strong O—H⋯O and N—H⋯O hydrogen bonds, together with water-mediated bridges, construct the primary chains; weaker C—H⋯O inter­actions inter­connect these chains into sheets; and finally, π–π inter­actions reinforce the packing to produce a robust three-dimensional supra­molecular architecture.

## 4. Database survey

While several examples utilizing 3-methyl­pyridine as a supra­molecular synthon have been documented in earlier studies (Trollip *et al.*, 2025 ▸; Yamada *et al.*, 2013 ▸), no corresponding crystallographic examples employing 5-nitro­isophthalic acid as a supra­molecular synthon were found in the available literature.

## 5. Synthesis and crystallization

3-Methyl­pyridine (1m*M*, 0.0913 g) and 5-nitro­isopthalic acid (1m*M*, 0.2111 g) were taken in an oven-dried round-bottom flask. Then 20 mL of water was added and the reaction mixture was refluxed at 373 K for 5h. The resulting solution was then cooled at room temperature and kept undisturbed for crystallization. After a few days, colourless needle-shaped good-quality crystals evolved from the solution.

## 6. Refinement

Crystal data, data collection parameters, and structure refinement details are compiled in Table 2 ▸. The H attached to aromatic carbon atoms were refined with the riding model. The HFIX 43 (one hydrogen on an *sp*^2^-hybriduzed carbon atom) command was used to add the C-bound hydrogen atoms (C1, C7, C3, C9, C10, C11, C12, C13). Subsequently, the hydrogen atoms on the methyl carbon (C14) of the 3-methyl­pyridinium cation were added with the help of HFIX 137 (three hydrogen atoms on *sp*^3^-hybridized carbon atom) command and the hydrogen on the nitro­gen atom (N2) of the pyridinium cation was found in a difference-Fourier map. The hydrogen atom on the –COOH group (O1) was fixed with HFIX 134 (one hydrogen O—H⋯H bonded). The hydrogen atoms associated with the water mol­ecule (O2) were identified from difference-Fourier maps and refined using the DFIX command.

## Supplementary Material

Crystal structure: contains datablock(s) I. DOI: 10.1107/S2056989026008091/dx2072sup1.cif

Structure factors: contains datablock(s) I. DOI: 10.1107/S2056989026008091/dx2072Isup2.hkl

Supporting information file. DOI: 10.1107/S2056989026008091/dx2072Isup3.cml

CCDC reference: 2579208

Additional supporting information:  crystallographic information; 3D view; checkCIF report

## Figures and Tables

**Table 1:** Hydrogen-bond geometry (Å, °)

*D*—H⋯*A*	*D*—H	H⋯*A*	*D*⋯*A*	*D*—H⋯*A*
C12—H12⋯O4	0.93	2.59	3.201 (3)	124
O1—H1⋯O2	0.92 (3)	1.64 (3)	2.5473 (19)	170 (3)
N2—H2⋯O3	0.89 (3)	1.77 (3)	2.643 (2)	171 (3)
O2—H2*A*⋯O4^i^	0.84 (2)	1.89 (2)	2.718 (2)	171 (3)
O2—H2*B*⋯O3^ii^	0.82 (2)	2.03 (2)	2.829 (2)	163 (3)

Symmetry codes: (i) 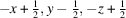; (ii) 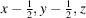.

**Table 2:** Experimental details

Crystal data	
Chemical formula	C_6_H_8_N^+^·C_8_H_4_NO_6_^−^·H_2_O
*M*_r_	322.27
Crystal system, space group	Monoclinic, *C*2/*c*
Temperature (K)	296
*a*, *b*, *c* (Å)	14.001 (4), 14.989 (4), 14.269 (4)
β (°)	94.550 (5)
*V* (Å^3^)	2985.2 (15)
*Z*	8
Radiation type	Mo *K*α
μ (mm^−1^)	0.12
Crystal size (mm)	0.21 × 0.18 × 0.15

Data collection	
Diffractometer	Bruker APEXII CCD
No. of measured, independent and observed [*I* > 2σ(*I*)] reflections	37111, 4407, 3029
*R*_int_	0.066
(sin θ/λ)_max_ (Å^−1^)	0.709

Refinement	
*R*[*F*^2^ > 2σ(*F*^2^)], *wR*(*F*^2^), *S*	0.069, 0.181, 1.07
No. of reflections	4407
No. of parameters	225
No. of restraints	2
H-atom treatment	H atoms treated by a mixture of independent and constrained refinement
Δρ_max_, Δρ_min_ (e Å^−3^)	0.37, −0.49

Computer programs: *APEX2* and *SAINT* (Bruker, 2012 ▸), *SHELXT* (Sheldrick, 2015*a* ▸), *SHELXL2019/3* (Sheldrick, 2015*b* ▸) and *OLEX2* (Dolomanov *et al.*, 2009 ▸).

## supplementary crystallographic information

### 3-Methylpyridinium 3-carboxy-5-nitrobenzoate monohydrate Crystal data

**Table:**

C_6_H_8_N^+^·C_8_H_4_NO_6_^−^·H_2_O	*F*(000) = 1344
*M**_r_* = 322.27	*D*_x_ = 1.434 Mg m^−^^3^
Monoclinic, *C*2/*c*	Mo *K*α radiation, λ = 0.71073 Å
*a* = 14.001 (4) Å	Cell parameters from 7138 reflections
*b* = 14.989 (4) Å	θ = 2.4–28.3°
*c* = 14.269 (4) Å	µ = 0.12 mm^−^^1^
β = 94.550 (5)°	*T* = 296 K
*V* = 2985.2 (15) Å^3^	Block, colorless
*Z* = 8	0.21 × 0.18 × 0.15 mm

### 3-Methylpyridinium 3-carboxy-5-nitrobenzoate monohydrate Data collection

**Table:**

Bruker APEXII CCD diffractometer	*R*_int_ = 0.066
φ and ω scans	θ_max_ = 30.3°, θ_min_ = 3.4°
37111 measured reflections	*h* = −19→19
4407 independent reflections	*k* = −21→21
3029 reflections with *I* > 2σ(*I*)	*l* = −20→20

### 3-Methylpyridinium 3-carboxy-5-nitrobenzoate monohydrate Refinement

**Table:**

Refinement on *F*^2^	Primary atom site location: structure-invariant direct methods
Least-squares matrix: full	Hydrogen site location: mixed
*R*[*F*^2^ > 2σ(*F*^2^)] = 0.069	H atoms treated by a mixture of independent and constrained refinement
*wR*(*F*^2^) = 0.181	*w* = 1/[σ^2^(*F*_o_^2^) + (0.085*P*)^2^ + 1.6623*P*] where *P* = (*F*_o_^2^ + 2*F*_c_^2^)/3
*S* = 1.07	(Δ/σ)_max_ < 0.001
4407 reflections	Δρ_max_ = 0.37 e Å^−^^3^
225 parameters	Δρ_min_ = −0.49 e Å^−^^3^
2 restraints	

### 3-Methylpyridinium 3-carboxy-5-nitrobenzoate monohydrate Special details

**Table:**

Geometry. All esds (except the esd in the dihedral angle between two l.s. planes) are estimated using the full covariance matrix. The cell esds are taken into account individually in the estimation of esds in distances, angles and torsion angles; correlations between esds in cell parameters are only used when they are defined by crystal symmetry. An approximate (isotropic) treatment of cell esds is used for estimating esds involving l.s. planes.

### 3-Methylpyridinium 3-carboxy-5-nitrobenzoate monohydrate Fractional atomic coordinates and isotropic or equivalent isotropic displacement parameters (Å^2^)

**Table:**

	*x*	*y*	*z*	*U*_iso_*/*U*_eq_	
O1	0.36724 (9)	−0.07423 (9)	0.32424 (11)	0.0527 (4)	
O2	0.21930 (10)	−0.17216 (10)	0.29414 (12)	0.0500 (4)	
O3	0.55819 (10)	0.36420 (9)	0.39569 (11)	0.0529 (4)	
O4	0.40090 (10)	0.34674 (10)	0.36377 (11)	0.0571 (4)	
O5	0.27850 (10)	0.04296 (10)	0.27574 (13)	0.0628 (4)	
O6	0.74673 (9)	0.09885 (12)	0.49234 (12)	0.0647 (5)	
O7	0.69029 (11)	−0.03206 (12)	0.46412 (14)	0.0735 (5)	
N1	0.68314 (10)	0.04850 (12)	0.46136 (11)	0.0457 (4)	
N2	0.50959 (13)	0.53448 (12)	0.38409 (12)	0.0471 (4)	
C1	0.52059 (11)	0.03101 (12)	0.38648 (11)	0.0352 (4)	
H1A	0.528922	−0.030520	0.388917	0.042*	
C2	0.43451 (10)	0.06861 (11)	0.34855 (11)	0.0334 (3)	
C3	0.42381 (11)	0.16076 (12)	0.34615 (11)	0.0348 (3)	
H3	0.366433	0.185404	0.321000	0.042*	
C4	0.48381 (12)	0.31673 (12)	0.37892 (12)	0.0401 (4)	
C5	0.59294 (10)	0.08824 (12)	0.42025 (11)	0.0357 (4)	
C6	0.49779 (11)	0.21682 (11)	0.38085 (11)	0.0349 (3)	
C7	0.58390 (11)	0.17971 (12)	0.41832 (11)	0.0372 (4)	
H7	0.634195	0.216028	0.441536	0.045*	
C8	0.35184 (11)	0.01167 (12)	0.31163 (13)	0.0383 (4)	
C9	0.47158 (15)	0.71004 (14)	0.36545 (14)	0.0517 (5)	
H9	0.457902	0.770547	0.359053	0.062*	
C10	0.56465 (14)	0.68319 (14)	0.39173 (13)	0.0480 (4)	
C11	0.58072 (14)	0.59327 (15)	0.40111 (14)	0.0492 (5)	
H11	0.642054	0.572838	0.419599	0.059*	
C12	0.41998 (14)	0.55942 (15)	0.35814 (14)	0.0518 (5)	
H12	0.372180	0.516842	0.346604	0.062*	
C13	0.39898 (15)	0.64855 (16)	0.34863 (15)	0.0548 (5)	
H13	0.336755	0.667193	0.331114	0.066*	
C14	0.6452 (2)	0.7490 (2)	0.4071 (2)	0.0827 (8)	
H14A	0.644178	0.789193	0.354609	0.124*	
H14B	0.705225	0.717712	0.412902	0.124*	
H14C	0.637817	0.782262	0.463557	0.124*	
H1	0.3152 (19)	−0.1097 (19)	0.3067 (18)	0.075 (8)*	
H2	0.530 (2)	0.479 (2)	0.3936 (19)	0.083 (9)*	
H2A	0.1873 (18)	−0.1635 (19)	0.2430 (14)	0.075 (8)*	
H2B	0.1809 (19)	−0.159 (2)	0.3326 (18)	0.092 (11)*	

### 3-Methylpyridinium 3-carboxy-5-nitrobenzoate monohydrate Atomic displacement parameters (Å^2^)

**Table:**

	*U*^11^	*U*^22^	*U*^33^	*U*^12^	*U*^13^	*U*^23^
O1	0.0311 (6)	0.0382 (7)	0.0865 (10)	−0.0059 (5)	−0.0099 (6)	0.0024 (7)
O2	0.0347 (7)	0.0438 (8)	0.0702 (10)	−0.0086 (6)	−0.0046 (7)	−0.0031 (7)
O3	0.0390 (7)	0.0399 (7)	0.0783 (10)	−0.0051 (5)	−0.0051 (6)	−0.0077 (6)
O4	0.0409 (7)	0.0478 (8)	0.0804 (10)	0.0050 (6)	−0.0088 (7)	−0.0075 (7)
O5	0.0370 (7)	0.0507 (8)	0.0956 (12)	−0.0054 (6)	−0.0263 (7)	0.0071 (8)
O6	0.0325 (7)	0.0790 (11)	0.0792 (10)	−0.0080 (7)	−0.0165 (6)	0.0071 (8)
O7	0.0457 (8)	0.0580 (10)	0.1124 (15)	0.0083 (7)	−0.0217 (8)	0.0134 (9)
N1	0.0265 (7)	0.0600 (11)	0.0494 (9)	−0.0013 (7)	−0.0037 (6)	0.0095 (7)
N2	0.0512 (9)	0.0401 (9)	0.0500 (9)	0.0037 (7)	0.0034 (7)	−0.0019 (7)
C1	0.0272 (7)	0.0382 (9)	0.0401 (8)	−0.0012 (6)	0.0015 (6)	0.0047 (6)
C2	0.0238 (7)	0.0402 (9)	0.0358 (8)	−0.0044 (6)	0.0000 (6)	0.0009 (6)
C3	0.0238 (7)	0.0408 (9)	0.0392 (8)	0.0002 (6)	−0.0011 (6)	−0.0003 (6)
C4	0.0371 (8)	0.0389 (9)	0.0435 (9)	−0.0010 (7)	−0.0020 (7)	−0.0047 (7)
C5	0.0226 (7)	0.0482 (10)	0.0359 (8)	−0.0004 (6)	−0.0003 (5)	0.0058 (7)
C6	0.0295 (7)	0.0384 (9)	0.0365 (8)	−0.0032 (6)	0.0010 (6)	−0.0019 (6)
C7	0.0274 (7)	0.0467 (10)	0.0368 (8)	−0.0067 (7)	−0.0011 (6)	−0.0001 (7)
C8	0.0264 (7)	0.0404 (9)	0.0472 (9)	−0.0048 (6)	−0.0018 (6)	0.0007 (7)
C9	0.0608 (12)	0.0431 (11)	0.0515 (11)	0.0076 (9)	0.0064 (9)	0.0019 (8)
C10	0.0490 (10)	0.0511 (11)	0.0442 (9)	−0.0054 (9)	0.0061 (8)	−0.0051 (8)
C11	0.0411 (9)	0.0563 (12)	0.0499 (10)	0.0074 (8)	0.0008 (7)	−0.0039 (9)
C12	0.0464 (10)	0.0548 (12)	0.0536 (11)	−0.0066 (9)	0.0001 (8)	−0.0025 (9)
C13	0.0440 (10)	0.0622 (13)	0.0573 (12)	0.0091 (9)	−0.0021 (8)	0.0054 (9)
C14	0.0750 (17)	0.0785 (19)	0.095 (2)	−0.0280 (15)	0.0090 (14)	−0.0142 (15)

### 3-Methylpyridinium 3-carboxy-5-nitrobenzoate monohydrate Geometric parameters (Å, º)

**Table:**

O1—C8	1.315 (2)	C3—H3	0.9300
O1—H1	0.92 (3)	C3—C6	1.394 (2)
O2—H2A	0.836 (17)	C4—C6	1.510 (3)
O2—H2B	0.822 (18)	C5—C7	1.377 (3)
O3—C4	1.268 (2)	C6—C7	1.395 (2)
O4—C4	1.248 (2)	C7—H7	0.9300
O5—C8	1.206 (2)	C9—H9	0.9300
O6—N1	1.223 (2)	C9—C10	1.387 (3)
O7—N1	1.212 (2)	C9—C13	1.379 (3)
N1—C5	1.475 (2)	C10—C11	1.371 (3)
N2—C11	1.337 (3)	C10—C14	1.502 (3)
N2—C12	1.333 (3)	C11—H11	0.9300
N2—H2	0.89 (3)	C12—H12	0.9300
C1—H1A	0.9300	C12—C13	1.372 (3)
C1—C2	1.400 (2)	C13—H13	0.9300
C1—C5	1.384 (2)	C14—H14A	0.9600
C2—C3	1.390 (2)	C14—H14B	0.9600
C2—C8	1.500 (2)	C14—H14C	0.9600
			
C8—O1—H1	114.3 (17)	C5—C7—H7	120.7
H2A—O2—H2B	102 (3)	C6—C7—H7	120.7
O6—N1—C5	118.06 (17)	O1—C8—C2	113.28 (14)
O7—N1—O6	123.18 (16)	O5—C8—O1	124.32 (16)
O7—N1—C5	118.75 (15)	O5—C8—C2	122.38 (16)
C11—N2—H2	111.7 (19)	C10—C9—H9	119.4
C12—N2—C11	122.44 (19)	C13—C9—H9	119.4
C12—N2—H2	125.9 (19)	C13—C9—C10	121.1 (2)
C2—C1—H1A	121.0	C9—C10—C14	121.8 (2)
C5—C1—H1A	121.0	C11—C10—C9	117.07 (19)
C5—C1—C2	117.96 (16)	C11—C10—C14	121.1 (2)
C1—C2—C8	121.58 (15)	N2—C11—C10	121.05 (18)
C3—C2—C1	119.83 (14)	N2—C11—H11	119.5
C3—C2—C8	118.58 (14)	C10—C11—H11	119.5
C2—C3—H3	119.5	N2—C12—H12	120.3
C2—C3—C6	120.99 (14)	N2—C12—C13	119.34 (19)
C6—C3—H3	119.5	C13—C12—H12	120.3
O3—C4—C6	116.71 (15)	C9—C13—H13	120.5
O4—C4—O3	124.70 (17)	C12—C13—C9	118.95 (19)
O4—C4—C6	118.55 (15)	C12—C13—H13	120.5
C1—C5—N1	117.90 (16)	C10—C14—H14A	109.5
C7—C5—N1	118.97 (14)	C10—C14—H14B	109.5
C7—C5—C1	123.13 (14)	C10—C14—H14C	109.5
C3—C6—C4	119.98 (14)	H14A—C14—H14B	109.5
C3—C6—C7	119.40 (16)	H14A—C14—H14C	109.5
C7—C6—C4	120.61 (14)	H14B—C14—H14C	109.5
C5—C7—C6	118.68 (14)		
			
O3—C4—C6—C3	−167.89 (16)	C2—C3—C6—C4	−178.98 (15)
O3—C4—C6—C7	13.0 (2)	C2—C3—C6—C7	0.2 (2)
O4—C4—C6—C3	14.0 (2)	C3—C2—C8—O1	−174.88 (15)
O4—C4—C6—C7	−165.10 (16)	C3—C2—C8—O5	3.8 (3)
O6—N1—C5—C1	−179.09 (16)	C3—C6—C7—C5	−0.3 (2)
O6—N1—C5—C7	0.0 (2)	C4—C6—C7—C5	178.82 (15)
O7—N1—C5—C1	−0.1 (2)	C5—C1—C2—C3	−0.3 (2)
O7—N1—C5—C7	178.97 (18)	C5—C1—C2—C8	−179.11 (15)
N1—C5—C7—C6	−178.88 (14)	C8—C2—C3—C6	179.00 (15)
N2—C12—C13—C9	0.6 (3)	C9—C10—C11—N2	1.0 (3)
C1—C2—C3—C6	0.1 (2)	C10—C9—C13—C12	−0.1 (3)
C1—C2—C8—O1	4.0 (2)	C11—N2—C12—C13	−0.2 (3)
C1—C2—C8—O5	−177.31 (18)	C12—N2—C11—C10	−0.6 (3)
C1—C5—C7—C6	0.2 (2)	C13—C9—C10—C11	−0.7 (3)
C2—C1—C5—N1	179.19 (14)	C13—C9—C10—C14	177.9 (2)
C2—C1—C5—C7	0.1 (2)	C14—C10—C11—N2	−177.5 (2)

### 3-Methylpyridinium 3-carboxy-5-nitrobenzoate monohydrate Hydrogen-bond geometry (Å, º)

**Table:**

*D*—H···*A*	*D*—H	H···*A*	*D*···*A*	*D*—H···*A*
C12—H12···O4	0.93	2.59	3.201 (3)	124
O1—H1···O2	0.92 (3)	1.64 (3)	2.5473 (19)	170 (3)
N2—H2···O3	0.89 (3)	1.77 (3)	2.643 (2)	171 (3)
O2—H2*A*···O4^i^	0.84 (2)	1.89 (2)	2.718 (2)	171 (3)
O2—H2*B*···O3^ii^	0.82 (2)	2.03 (2)	2.829 (2)	163 (3)

Symmetry codes: (i) −*x*+1/2, *y*−1/2, −*z*+1/2; (ii) *x*−1/2, *y*−1/2, *z*.
